# Acute Effects of Subthalamic Deep Brain Stimulation on Motor Outcomes in Parkinson's Disease; 13 Year Follow Up

**DOI:** 10.3389/fneur.2019.00689

**Published:** 2019-06-26

**Authors:** Haiyan Zhou, Linbin Wang, Chencheng Zhang, Xian Qiu, Wei Hu, Odin van der Stelt, Adolfo Ramirez-Zamora, Dianyou Li

**Affiliations:** ^1^Department of Neurology, Ruijin Hospital, Shanghai Jiao Tong University School of Medicine, Shanghai, China; ^2^Department of Functional Neurosurgery, Ruijin Hospital, Shanghai Jiao Tong University School of Medicine, Shanghai, China; ^3^University of Florida Center for Movement Disorders and Neurorestoration, Gainesville, FL, United States; ^4^Independent Researcher, Amsterdam, Netherlands

**Keywords:** early-onset Parkinson's disease, motor symptoms, deep brain stimulation, subthalamic nucleus, long-term treatment

## Abstract

**Objective:** Deep brain stimulation of the Subthalamic nucleus (STN-DBS) is a safe and well-established therapy for the management of refractory motor symptoms in Parkinson's disease (PD). Marked improvement in axial symptoms has been reported in the short term with STN-DBS but questions remain regarding the long-term efficacy of this intervention. We assessed the acute ON and OFF effects of STN-DBS in PD patients who have been treated with STN-DBS for over a decade.

**Methods:** We assessed 11 patients with early-onset PD (9 men, 2 women; mean age, 57.1 ± 7.2 y; mean age at illness onset, 38.9 ± 7.5 y) managed with long-term bilateral STN-DBS (mean treatment duration, 13.4 ± 1.3 y). Motor symptoms were assessed by means of the Unified Parkinson's Disease Rating Scale (UPDRS)-III, Timed Up and Go test (TUG), and Hoehn-Yahr scale. Motor assessments in the medication ON and OFF states with stimulation ON and OFF conditions were documented and video recorded.

**Results:** Patients showed a significant improvement in motor symptoms both in the off-medication and on-medication state by a 54% reduction (off-medication/on-stimulation vs. off-medication/off-stimulation) and a 48% reduction (on-medication/on-stimulation vs. on-medication/off-stimulation) in the total UPDRS-III score. Specifically, improvement in axial symptoms (off-medication: 51% reduction; on-medication: 44% reduction), including gait but not posture. Similarly, STN-DBS reduced TUG scores (off-medication: 70% reduction; on-medication: 47% reduction).

**Conclusions:** On stimulation long-term, bilateral STN-DBS can improve appendicular and axial symptoms of patients with early-onset PD in the acute setting.

## Introduction

Parkinson's disease (PD) is a neurodegenerative disease characterized by progressive deterioration of motor function (1). The disease typically affects people older than 50 years of age, but some individuals might develop PD at a younger age (2). Current medications can provide clear symptomatic remission but patients with longer disease duration can develop motor complications and fluctuations. In addition, axial symptoms unresponsive to medications (e.g., impairments of speech, gait, balance, or posture) become increasingly evident as the disease progresses over time.

Deep brain stimulation of the Subthalamic nucleus (STN-DBS) is a safe and effective treatment for management of motor complications in patients with PD, providing excellent symptom control in the short- and long-term (3–9). By contrast, the impact of STN-DBS on the later occurring axial symptoms in PD remains inconclusive (10–12). Symptoms are usually multifactorial and determining the contribution of STN-DBS to actual reduction of disability is challenging, particularly in patients with heterogeneous disease progression. It is also possible that disease age of onset might impact the effect of STN-DBS on axial symptoms related to disease progression.

In this report, we aimed to assess the acute ON and OFF effects of STN-DBS on motor symptoms in a cohort of early-onset PD patients treated with STN-DBS for an average of 13 years. From a clinical perspective, our results should provide patients and clinicians with additional information about the risks and benefits of long-term STN-DBS treatment for PD.

## Materials and Methods

### Participants

Study participants consisted of 11 patients (9 men and 2 women) with early-onset PD (Supplementary Table 1). The patients were recruited from the Departments of Neurology and Functional Neurosurgery at Ruijin Hospital, Shanghai Jiao Tong University School of Medicine (Shanghai, China), using the following inclusion criteria: (1) idiopathic PD, according to the United Kingdom Parkinson's Disease Society Brain Bank Clinical Diagnostic Criteria; (2) use of a bilateral STN-DBS implant for more than 10 years; (3) onset of PD at or before 50 years of age; and (4) disease duration of 7 years or less at the time of surgery. Patients were excluded if they had unstable vital signs or medical and psychiatric comorbidities at the time of the study.

This cohort study was registered in the Clinical Trial Registry (ClinicalTrials.gov) (NCT03230331). The study protocol was approved by the Ruijin Hospital Ethics Committee of Shanghai Jiao Tong University School of Medicine (Shanghai, China). The study reported was conducted in accordance with the Declaration of Helsinki. All participants provided written informed consent.

### Clinical Assessments

The primary outcomes were defined as the change in the Unified Parkinson's Disease Rating Scale (UPDRS)- part III, the Timed Up and Go (TUG) test and the Hoehn-Yahr stage. UPDRS-III was used to assess the severity of motor symptoms, TUG test was used to assess gait and freezing of gait (FOG), and the Hoehn-Yahr stage to measure the level of disability and progression.

Stimulation parameters at each visit and adverse events were recorded. The total electrical energy delivered in 1 s (TEED_1s_) at last follow-up was calculated according to the following formula: [(Voltage^2^ × Frequency × PW)/Impedance] × 1 s (13).

Motor assessments were video recorded and scored by a rater blinded to the patient's study status and stimulation settings. Assessments were obtained after overnight withdrawal of dopaminergic drugs (off-medication state) and 45 min after administration of regular antiparkinsonian medication (on-medication state) with the STN-DBS turned on (stimulation-on condition) and repeated 1 h later with the stimulator turned off (stimulation-off condition) (14).

### Surgical Technique

The neurosurgical procedure was performed as previously described (15). The intended target coordinates were determined on the basis of 1.5 T magnetic resonance imaging (MRI), then the electrodes (model 3387–40, 7428, Medtronic, Minneapolis, MN, USA) were stereotactially implanted into the bilateral STN under local anesthesia. Intraoperative macroelectrode stimulation was used in confirming the target position. The DBS lead and programmable pulse generator (IPG) [bilateral Itrel® II, unilateral Kinetra; Medtronic] were connected using an extension wires (7482, Medtronic) and implanted subclavicularly under general anesthesia. IPG programming was initiated on the following day. Electrical parameters (voltage, pulse width, and frequency) were subsequently adjusted using standard DBS programmer (7532, 8840 neurological programmer; Medtronic). Post-operative imaging confirmed satisfactory placement of DBS leads.

### Statistical Analysis

The differences between the STIM ON and STIM OFF conditions, and stimulation parameters between different follow-up visits were compared by non-parametric Wilcoxon signed-ranks tests. All statistical analyses were conducted using Statistical Package for the Social Sciences for Windows (version 18.0; IBM, USA). A two-sided *p* < 0.05 was considered statistically significant. Significance-levels were adjusted using False Discovery Rate (FDR) correction to account for multiple testing.

## Results

### Patient Characteristics

A total of 11 patients (9 men and 2 women) with early-onset PD were enrolled. Patients were between 43 and 68 years of age (mean age at PD onset: 38.9 ± 7.5 years; mean age at surgery: 43.8 ± 8.7 years) and had received continuous STN-DBS treatment for an average duration of 13.4 ± 1.3 years at study entry.

Stimulation was delivered by a non-rechargeable Medtronic pulse generator (model 7428 Kinetra, or 7426, Soletra™), and batteries were replaced 3.9 ± 1.3 times with an average battery span of 4.1 ± 1.3 years. At the last replacement, all patients received new generation of Medtronic Activa™ pulse generator (model 37602 or 37612).

At last follow up (average of 13 years) dopaminergic medications were significantly reduced by 53% (Levodopa equivalent daily dose; baseline: 750 ± 224 mg/day; 13-year post-surgery: 356 ± 397 mg/day). Interestingly, two patients stopped dopaminergic medication due to side effects (hallucinations) and appropriate motor control and two additional patients required <100 mg levodopa per day to minimize dyskinesia.

### Motor Outcomes

Patients showed a significant improvement in motor symptoms both in the off-medication and on-medication state by a 54% reduction (off-medication/on-stimulation vs. off-medication/off-stimulation) and a 48% reduction (on-medication/on-stimulation vs. on-medication/off-stimulation) in the total UPDRS-III score (Table 1). Specifically, STN-DBS markedly controlled tremor (off-medication: 72% reduction; on-medication: 69% reduction) and rigidity (off-medication: 72% reduction; on-medication: 66% reduction), bradykinesia (off-medication: 45% reduction; on-medication: 40% reduction), and overall level of axial symptoms (off-medication: 51% reduction; on-medication: 44% reduction) including gait (off-medication: 56% reduction; on-medication: 50% reduction) but not posture (off-medication: 38% reduction; on-medication: 27% reduction). Similarly, STN-DBS impacted the patients' functional mobility, showing a clear reduction in TUG scores (off-medication: 70% reduction; on-medication: 47% reduction) and time percentage spend in FOG (off-medication: 54% reduction; on-medication: 58% reduction).

**Table 1:** Motor symptom severity of patients (*N* = 11) before and after STN-DBS^*^.

	**Med off**		**Med on**		***P*****-value**	
	**Stim off**	**Stim on**	**Stim off**	**Stim on**	**Med off**	**Med on**
**UPDRS III**						
Total	58.2 ± 19.9	26.8 ± 14.1	51.1 ± 22.7	26.8 ± 14.1	0.003	0.003
Tremor *(item 20, 21)*	3.9 ± 3.7	1.1 ± 2.1	2.9 ± 3.4	0.9 ± 1.9	0.018	0.018
Rigidity *(item 22)*	12.6 ± 5.1	3.6 ± 3.3	10.4 ± 6.2	3.5 ± 3.4	0.003	0.005
Bradykinesia *(item 19, 23–26,31)*	31.1 ± 9.0	17.0 ± 8.8	28.3 ± 9.6	17.0 ± 8.8	0.003	0.005
**Axial symptoms** ***(item 18, 27–30)***	10.6 ± 5.0	5.2 ± 2.5	9.3 ± 5.4	5.2 ± 2.5	0.003	0.008
Gait *(item 29)*	2.5 ± 1.3	1.1 ± 0.9	2.2 ± 1.4	1.1 ± 0.9	0.003	0.01
Posture *(item 28)*	1.3 ± 0.7	0.8 ± 0.8	1.1 ± 0.5	0.8 ± 0.8	0.102	0.083
**Timed up and go^N^a^^**						
Total time in seconds	33 ± 48	10 ± 4	18.6 ± 14.3	9.9 ± 3.7	0.017	0.017
Time percentage spend in FOG, %	27.3 ± 29.8	12.6 ± 15.7	27.3 ± 29.8	11.4 ± 13.3	0.018	0.018
**Hoehn-Yahr stage**	3.3 ± 1.0	2.5 ± 0.6	3.2 ± 1.0	2.5 ± 0.6	0.007	0.017

* *Data represent mean and standard deviation. The two stimulation conditions differed significantly from each other at p <0.05 for all variables in both medication-on and -off state, except Posture (item 28), as assessed by two-tailed Wilcoxon signed-ranks tests. Significance-levels were adjusted using False Discovery Rate (FDR) Correction to account for multiple testing*.

*STN, subthalamic nucleus; DBS, deep brain stimulation; UPDRS, United Parkinson Disease Rating Scale*.

*N^a^ = 7, 4 of 11 patients can walk only with stimulation in both medication -on and -off state*.

### Stimulation Parameters

Monopolar stimulation with single or double unipolar configuration was utilized in 90 percent of patients. The mean (SD) stimulation voltage used for stimulation was significantly increased over time, whereas frequency of stimulation was reduced. By contrast, pulse width kept steady during stimulation, except for the first visit (Table 2). At last follow-up, the total electrical energy delivered by deep brain stimulation of the STN was 343.6 ± 118.8 μW.

**Table 2:** Stimulation parameters at follow up visits 1 month, 1 year, 5 years, 8 years, and 13 years after surgery^*^.

**Stimulation parameters**	**1 month**	**1 year**	**5 year**	**8 year**	**13 year**
Amplitude (V)	2.15 ± 0.16	2.51 ± 0.25^a^	2.84 ± 0.40^a^^,^^b^	3.00 ± 0.42^a^^,^^b^^,^^c^	3.11 ± 0.34^a^^,^^b^^,^^c^
Pulse width (μs)	60 ± 0	74 ± 16^a^	82 ± 24^a^	82 ± 24^a^	92 ± 18^a^^,^^b^
Rate (Hz)	165 ± 10	163 ± 12	152 ± 10^a^^,^^b^	140 ± 18^a^^,^^b^^,^ ^c^	128 ± 26^a^^,^^b^^,^^c^

* *Data represent mean and standard deviation*.

a *p significant compared with 1 month after surgery*.

b *p significant compared with 1 year after surgery*.

c *p significant compared with 5 years after surgery. p <0.05 was considered as significant difference, using two-tailed Wilcoxon signed-ranks tests*.

### Adverse Events

There were no hardware related or surgical related complications in this group of patients. Four patients had stimulation induced dyskinesia during the first 3 months of stimulation that controlled by reducing the stimulation or medications. Weight gained of ~8 kg was noted at 1 year in 3 patients.

## Discussion

In this study, we assessed the acute ON and OFF effects of STN-DBS in early-onset PD patients treated with STN-DBS for an average of 13 years. We included only patients with early-onset PD (defined by diagnosis of PD before age 50) in an attempt to enroll a relatively homogeneous group of patients. Acute STN-DBS treatment showed a statistically and clinically improvement in the cardinal motor symptoms of PD and we observed a persistent benefit axial symptoms including gait difficulties. The procedure was well-tolerated and side effects resolved with programming or medication adjustments. Our study suggests in early-onset PD patents, STN-DBS can provide sustained, discernible motor benefits. Additionally, axial PD symptoms may improve at long-term follow-up. However, additional research is needed as our sample size is small.

The observed effects of STN-DBS on the axial symptoms in this study are consistent with some previous reports suggesting that long-term STN-DBS treatment might continue to manage axial symptoms over the disease course (11). However, there are also negative outcome reports in terms of long-term STN stimulation for axial symptoms. The timing of surgery might be an important factor. Castrioto et al. (3) found worsening effects of STN-DBS on axial signs after 10 years' stimulation (3). Even with similar age of PD onset [39.6 ± 6.6 years (3) vs. 38.9 ± 7.5 years], the age at surgery in their report was much later than our cohort [52.9 ± 7.9 years (3) vs. 43.8 ± 8.7 years].

Adequate, expert programming is clearly relevant to optimize the long-term effects of STN-DBS, including strategies to minimize side effects like low frequency stimulation for postural instability, gait difficulties and dysphasia (16). During DBS programming sessions, electrical charge and voltage were progressively increased according to changes in their clinical status, whereas the pulse width of stimulation did not require major adjustments over the long-term course of the treatment. Lower frequency stimulation (80–145 Hz) was used later in the course of the disease to manage the axial symptoms as we reported in a preview study (15).

This study has several limitations that should be considered when interpreting the results. First, the study recruited a small number of patients, without case controls or randomization. The two STN-DBS conditions were administrated to the patients in a fixed order, and not in a random or counterbalanced order. This suggest that the observed STN-DBS motor effects could have been confounded by persistent effects and limited washout period. Additionally, we were unable to obtained specific baseline UPDRS scores prior to STN-DBS treatment, limiting individual long term assessments. Finally, as patients in the study had earlier onset of motor symptoms, the results might not be representative of the entire PD population. Although encouraging, additional randomized, blinded research is needed.

## Ethics Statement

This study was carried out in accordance with the recommendations of name of guidelines, name of committee with written informed consent from all subjects. All subjects gave written informed consent in accordance with the Declaration of Helsinki. The protocol was approved by the name of committee.

## Author Contributions

HZ and LW has contributed to study design, data analysis, and the writing of this research report. OvdS, XQ, and CZ contributed to the writing of this report. AR-Z, WH, and DL contributed to study design and the writing of this report.

### Conflict of Interest Statement

CZ and DL received honoraria and travel expenses from Medtronic Inc. (Minnesota, USA), PINS Medical Co., Ltd. (Beijing, China), and SceneRay Corp., Ltd. (Suzhou, China). AR-Z received financial compensation for consulting services to Medtronic Inc. (Minnesota, USA), Wilsons therapeutics, and Bracket. The remaining authors declare that the research was conducted in the absence of any commercial or financial relationships that could be construed as a potential conflict of interest.

## Abbreviations

STN: Subthalamic nucleus
DBS: deep brain stimulation
PD: Parkinson's disease
UPDRS: Unified Parkinson's Disease Rating Scale
TUG: Timed Up and Go test
PIGD: postural instability and gait difficulty.
