# Supplementary material for: Acute Effects of Subthalamic Deep Brain Stimulation on Motor Outcomes in Parkinson's Disease; 13 Year Follow Up
Source: Front Neurol. 2019 Jun 26;10:689. doi: 10.3389/fneur.2019.00689 (PMC6606731; doi:10.3389/fneur.2019.00689)
Supplement: Supplementary file 1 [file Data_Sheet_1.PDF]

Supplementary Table 1. Demographic and clinical characteristics of patients (N = 11)*

Demographic/clinical variable	
Gender (M:F)	9:2
Age at follow-up (years)	57.1 ± 7.2 (43-68)
Disease duration at follow-up (years)	18.2 ± 1.9 (14-21)
Age at Parkinson's disease onset(years)	38.9 ± 7.5 (24-50)
Age at STN-DBS surgery (years)	43.8 ± 8.7 (28-56)
Disease duration at STN-DBS onset (years)	4.9 ± 1.2 (2-6)
STN-DBS treatment duration (years)	13.4 ± 1.28 (12-15)

* Data represent mean ± standard deviation (range), unless indicated otherwise. STN, subthalamic nucleus; DBS, deep brain stimulation.
